# Pan-Genome Analysis of Campylobacter: Insights on the Genomic Diversity and Virulence Profile

**DOI:** 10.1128/spectrum.01029-22

**Published:** 2022-09-07

**Authors:** Chaofang Zhong, Bingpeng Qu, Gang Hu, Kang Ning

**Affiliations:** a College of Environmental and Life Sciences, Nanning Normal University, Nanning, Guangxi, China; b Key Laboratory of Molecular Biophysics of the Ministry of Education, Hubei Key Laboratory of Bioinformatics and Molecular-Imaging, Department of Bioinformatics and Systems Biology, College of Life Science and Technology, Huazhong University of Science and Technology, Wuhan, Hubei, China; Brown University

**Keywords:** *Campylobacter*, pan-genome, pathogenicity, virulence factor, antibiotic resistance

## Abstract

The genus Campylobacter contains pathogens that cause bacterial gastroenteritis in humans and animals. Despite large-scale sequencing efforts to raise clinical awareness of Campylobacter, little is known about the diversity and functions of virulence factors. Here, we constructed the pan-genome of Campylobacter using 39 representative genomes, elucidating their genetic diversity, evolutionary characteristics, and virulence and resistance profiles. The Campylobacter pan-genome was open and showed extensive genome variability, with high levels of gene expansion and contraction as the organism evolved. These Campylobacter members had diverse virulence gene content, and six potential core virulence genes (*porA*, *PEB4*, *cheY*, *htrB*, *Cj1135*, and *kpsF*) have been identified. The conserved mechanisms for Campylobacter pathogenicity were related to adherence, motility, and immune modulation. We emphasized the relative importance of variable virulence genes. Many virulence genes have experienced expansion or contraction in specific lineages, which may be one of the factors causing differences in the content of virulence genes. Additionally, these Campylobacter genomes have a high prevalence of the *cmeA* and *cmeC* genes, which are linked to the CmeABC pump and contribute to multidrug resistance. The genomic variations, core and variable virulence factors, and resistance genes of Campylobacter characterized in this study would contribute to a better understanding of the virulence of Campylobacter and more effective use of candidates for drug development and prevention of Campylobacter infections.

**IMPORTANCE** Pathogenic members of the genus Campylobacter are recognized as one of the major causative agents of human bacterial gastroenteritis. This study revealed the pan-genome of 39 Campylobacter species, provided the most updated reconstruction of the global virulence gene pool of 39 Campylobacter species, and identified species-related virulence differences. This study highlighted the basic conserved functionality and specificity of pathogenicity that are crucial to infection, which was critical for improving the diagnosis and prevention of Campylobacter infections.

## INTRODUCTION

Campylobacter is a rod-shaped bacterium, several species of which are important pathogens for humans and animals (1, 2), and some exhibit a high degree of host specificity or host preference (3, 4). The recent application of whole-genome sequencing (WGS) has emphasized the importance of Campylobacter species in human and animal infections (5, 6). Infection with pathogenic Campylobacter species, such as C. jejuni and C. coli, is one of the leading causes of gastroenteritis in humans worldwide (7, 8). These pathogenic Campylobacter members have also been proven to cause bacterial foodborne illness and rapidly colonize and spread in the host (3, 9, 10), thereby increasing their threat to human health and posing rising economic losses worldwide (11). Thus, the pathogenic mechanisms of Campylobacter have attracted worldwide attention. Studies on virulence factors of Campylobacter have shown that some Campylobacter species contain virulence factors, such as flagella and toxins, which are relevant to pathogenesis (12, 13). However, emerging Campylobacter species are reported to have diverse pathogenic mechanisms and differ in pathogenicity and host specificity (14, 15). Specifically, different Campylobacter species exhibit some unique characteristics. For example, C. jejuni and C. coli are the most common causative agents of human bacterial gastroenteritis (13), but C. sputorum and C. mucosalis infections are rarely reported in humans (16), and the reason for this difference is still unclear.

The genetic diversity of the pathogenic factors of Campylobacter is an important cause of differing pathogenicity (17). Much research has been invested to identify species-unique genes of Campylobacter and genes of different pathogenicity (18, 19). With the application of whole-genome sequencing technologies, diverse Campylobacter species have been sequenced in recent years (20, 21). Previous studies have discovered genes functionally associated with pathogenic mechanisms in Campylobacter species (22, 23), but the pictures of genetic diversity for pathogenic regions across different species are still incomplete, especially those virulence factors that are unique to a species. In the case of unknown population-specific or individual-specific virulence factors and pathogenic mechanisms, clinical treatment and medication for different Campylobacter infections may be difficult. In addition, inaccurate Campylobacter species identification can lead to misdiagnosis. Therefore, to promote the identification, functional identification, and disease diagnosis of Campylobacter, it is necessary to carry out accurate classification and detailed analysis of pathogenic factors for dozens of Campylobacter species.

The pan-genome refers to the pool of genetic material that is present in a group of bacteria (24). The pan-genome analysis has provided new insights into interspecies differentiation and whole sets of genes shared among a group of bacteria (25, 26). The application of pan-genome analysis to pathogenic Campylobacter will shed light on infection mechanisms and core genes or unique genes. In particular, the pan-genomic analysis of C. hyointestinalis subspecies has identified diverse patterns associated with the host and demonstrated the utility of pan-genomics in the biological discovery of Campylobacter (27). Capturing unique genes is particularly relevant for Campylobacter because the main determinants of pathogenicity are usually encoded by genes that are not shared by all species. Analysis of 96 genome sequences derived from C. coli and C. jejuni showed that each of their genomes has unique cohesive features (28). The establishment of a comprehensive Campylobacter pan-genome will help to accurately identify species and analyze the functional adaptation of individual species. In addition, assessment of the pan-genome profiles allowed the most updated reconstruction of the global virulence pool of Campylobacter based on currently available data, which will further promote the diagnosis and treatment of Campylobacter infections.

Currently, we are still far from obtaining a comprehensive pattern on the evolution and pathogenicity of Campylobacter. Fortunately, the sequenced and assembled genomes for 39 Campylobacter species were reported and made available in public databases, which enabled understanding of their genomic diversity and pathogenic profiles. Here, we performed a pan-genome study for 39 representative members of the genus Campylobacter that are publicly available. These 39 representative high-quality genomes were full-genome representations and were isolated mainly from humans, animals, and the natural environment. The genomic information, isolation source, geographical location, and collection date are provided in Table S1 in the supplemental material. We estimated both the sizes of the pan-genome and core genome and illustrated the distribution of core and noncore genes. We concentrated on identifying the virulence factors and emphasized the influence of gene expansion and contraction in the evolution of Campylobacter virulence. This pan-genome resource of Campylobacter will facilitate the development of intervention strategies for the prevention and treatment of Campylobacter infections.

## RESULTS AND DISCUSSION

### Pan-genome size of 39 Campylobacter species.

**FIG 1:**
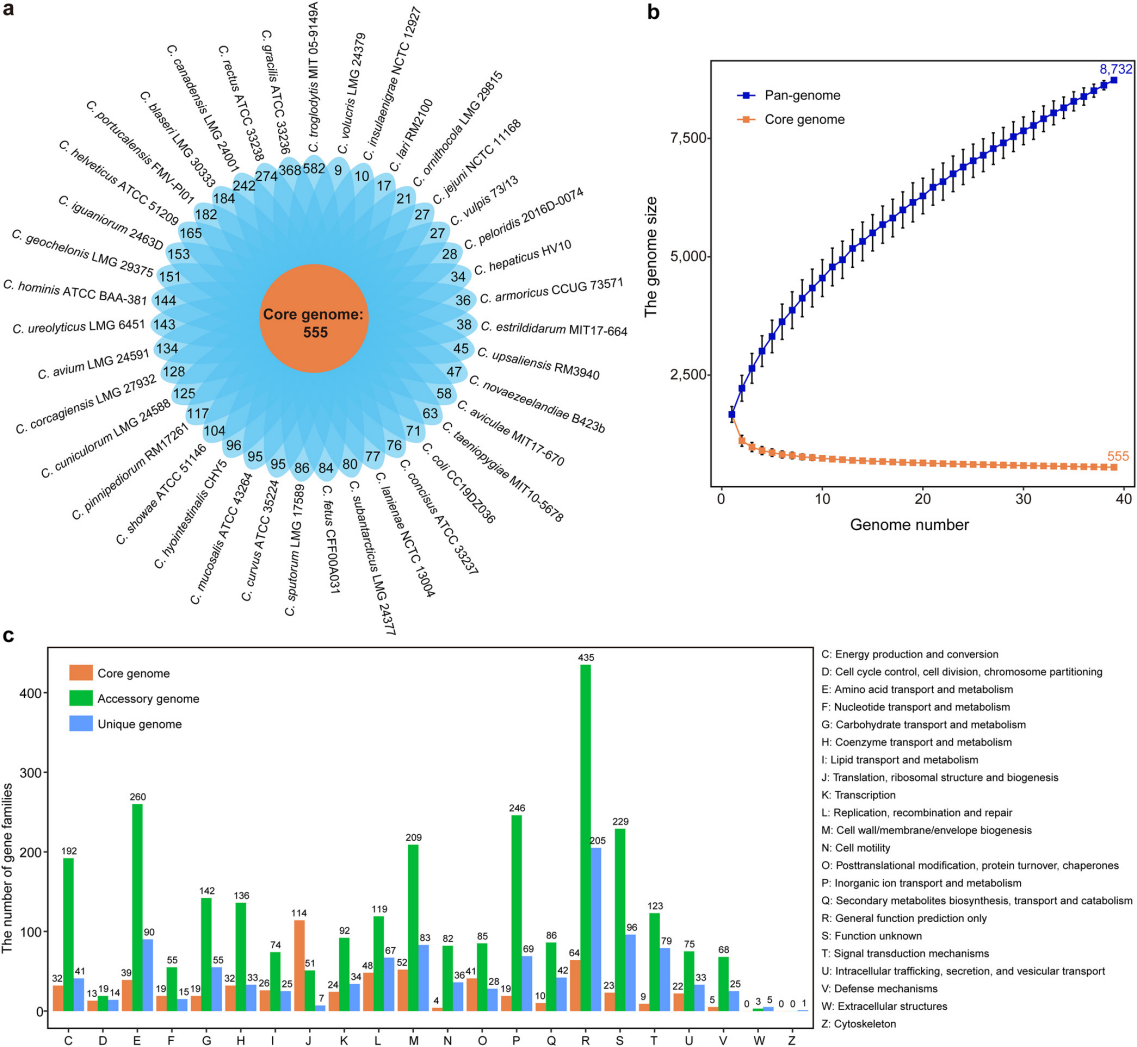
Pan-genome structure and the gene functional distribution of Campylobacter. (a) Core genome size and the distributions of unique genes in each Campylobacter species. (b) The cumulative sizes for the pan-genome (blue) and core genome (orange). (c) Distribution of COG categories in Campylobacter core, accessory, and unique genomes.

To capture the entire genomic diversity of Campylobacter, we selected 39 species with complete genomes (Table S1 in the supplemental material) for the pan-genome study, each of which represented the genome with the highest assembly level when multiple strain sequences were available for a Campylobacter species. To understand the pan-genome of Campylobacter more deeply, 67,533 protein-coding genes obtained from the selected 39 sequenced Campylobacter genomes were clustered by OrthoMCL. We identified a total of 8,732 gene families, of which 555 (6.36%) were shared by all 39 analyzed genomes (Fig. 1a), constituting the core genome (gene families present in all 39 analyzed genomes). The accessory genome included 3,761 (43.07%) gene families shared by at least two species, and 4,416 (50.57%) unique gene families were found in only one strain, constituting the unique genome. This high proportion of unique genome (50.57%) further supports the intragenus heterogeneity of Campylobacter and confirmed the previously observed highly diverse nature of this bacteria at the whole-genome level (23, 27, 28). Among these Campylobacter species, C. troglodytis MIT 05-9149A isolated from chimpanzees (29) and C. gracilis ATCC 33236 isolated from human oral samples (30) possessed more unique genes, with 582 and 368 genes, respectively (Fig. 1a). The variable genes (accessory genes and unique genes) account for about 93.64% of the whole pan-genome, signifying the high level of genetic diversity in the members of Campylobacter and that the genus is a very heterogeneous population.

The core genome and pan-genome sizes of 39 analyzed Campylobacter genomes were estimated to be 555 and 8,732 gene families, respectively. The number of genomes examined greatly influences the size of the core genome and pan-genome. The curves of the core genome and pan-genome size of these Campylobacter genomes with the increase of the number of genomes showed that the pan-genome size increased almost exponentially with the number of genomes, while the core genome size was being narrowed (Fig. 1b). When the number of added genomes reached 39, the size of the pan-genome still increased. The measured size of the pan-genome was well fit with a power law function *y* = *Ax^b^*, where *A* is 1,600.852 and *b* is 0.459. The core genome curve was well described by a decaying power function *y* = *Ax^−b^*, where *A* is 1,349.841 and *b* is 0.248. Estimates of pan-genome and core genome sizes did not level out, and it appears that they might still be influenced by the inclusion of new genome sequences, suggesting that Campylobacter has an open pan-genome and core genome. Thus, the complete pan-genome of the Campylobacter genus was likely substantially larger than that estimated by these 39 genomes. This trend continued with the addition of multiple genomes for these 39 genomes; for example, when adding to the 251 genomes, the pan-genome size still increased slightly, while the core genome decreased to 252 gene families (Fig. S1). This result signified a high level of intraspecific genetic diversity in Campylobacter and indicated that a single reference genome does not represent the diversity within a species. As a result, as more species are identified and analyzed, the global gene repertoire of the Campylobacter genus would change considerably in the future.

### Functional features of the Campylobacter pan-genome.

To clarify the functional characteristics of the Campylobacter genome, the clusters of orthologous groups (GOG) analysis was used to analyze whether core, accessory, and unique genomes in the pan-genome had distinct COG profiles. The functional categories of the genes were assigned to core, accessory, and unique classes, and the results showed that the gene families in the Campylobacter core genome were enriched for genes involved in “translation, ribosomal structure, and biogenesis” (J) (Fig. 1c). The overall proportion of genes involved in “translation, ribosomal structure, and biogenesis” (J) in the core genome was 20.54% (114/555), whereas that in the accessory and unique genomes was 1.36% (51/3,761) and 0.16% (7/4,416), respectively. Therefore, the COG analysis results highlighted that more core genes perform fundamental housekeeping functions than accessory genes and unique genes. Considering the open state of the Campylobacter pan-genome, the results of the COG enrichment analysis of Campylobacter accessory genes and unique genes, especially “amino acid transport and metabolism” (E), “replication, recombination, and repair” (L), “cell wall/membrane/envelope biogenesis” (M), and “inorganic ion transport and metabolism” (P) (Fig. 1c), were consistent with the perspective that larger genomes tend to accumulate functions to enable organisms to achieve a higher degree of ecological diversification (31). In addition, the virulence-associated functional categories, such as the functional categories of “cell motility” (N), “inorganic ion transport and metabolism” (P), “signal transduction mechanisms” (T), and “defense mechanisms” (V) (32, 33), were prominently represented in the variable component of the pan-genome, which might contribute to Campylobacter pathogenicity.

### Gene family expansion and contraction for Campylobacter.

**FIG 2:**
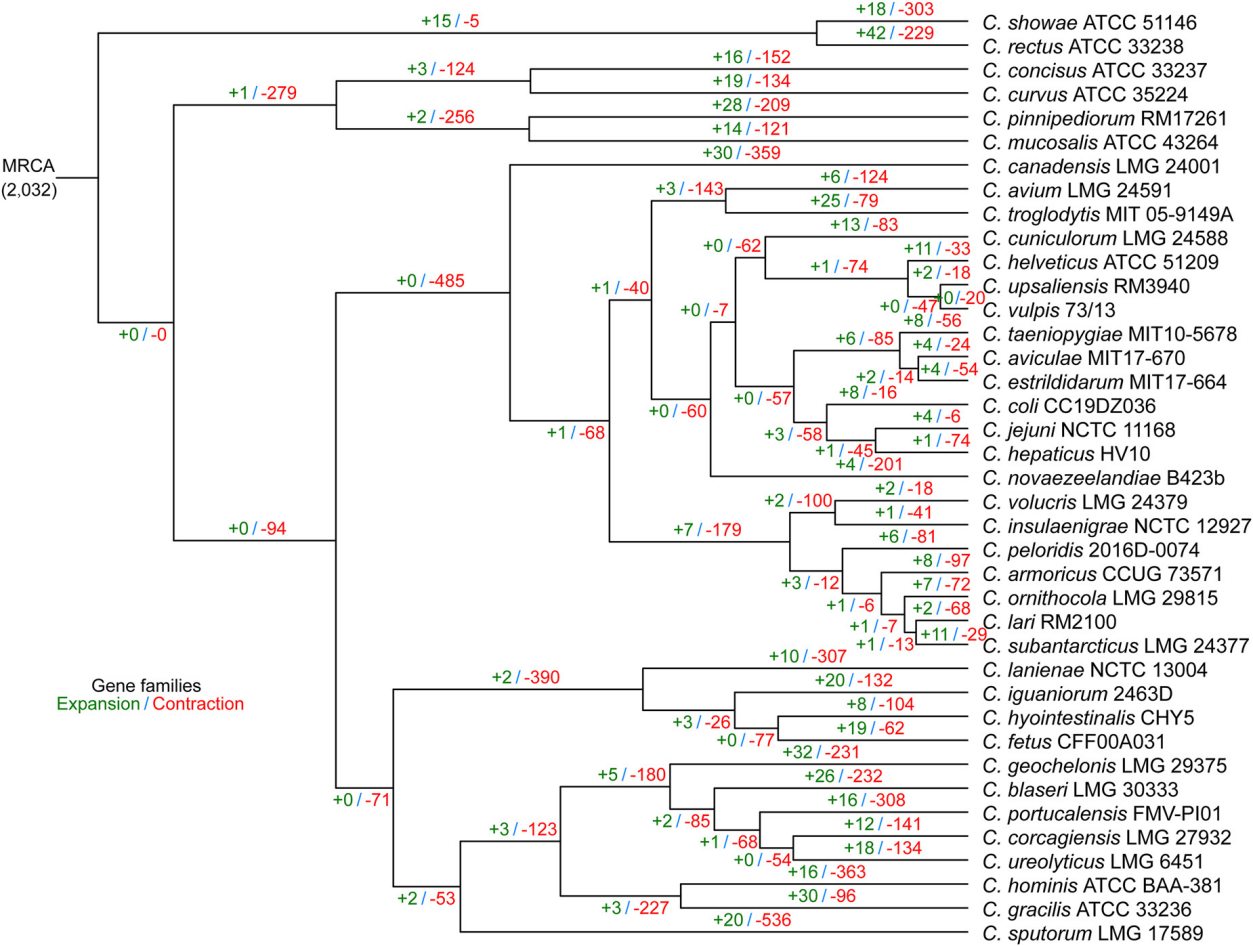
Expansion (green) and contraction (red) of gene families in each Campylobacter lineage. The tree was based on the concatenated sequences of 539 core genes. MRCA, most recent common ancestor.

For the purpose of analyzing the evolutionary process of Campylobacter, a comprehensive phylogenetic inference was determined for all 39 Campylobacter species. A phylogenetic tree based on 539 single-copy core genes was created. Phylogenetic analysis based on single-copy core genes supported a close relationship of C. showae ATCC 51146 and C. rectus ATCC 33238, which represented the most ancient lineages of Campylobacter (Fig. 2). C. jejuni NCTC 11168 as the most important pathogen for humans was more closely related to C. hepaticus HV10, followed by C. coli CC19DZ036. This result was consistent with previous pan-genome studies that found C. jejuni and C. coli to be closely related sister species (28, 34), and it expanded the evolutionary relationship between more species.

**FIG 3:**
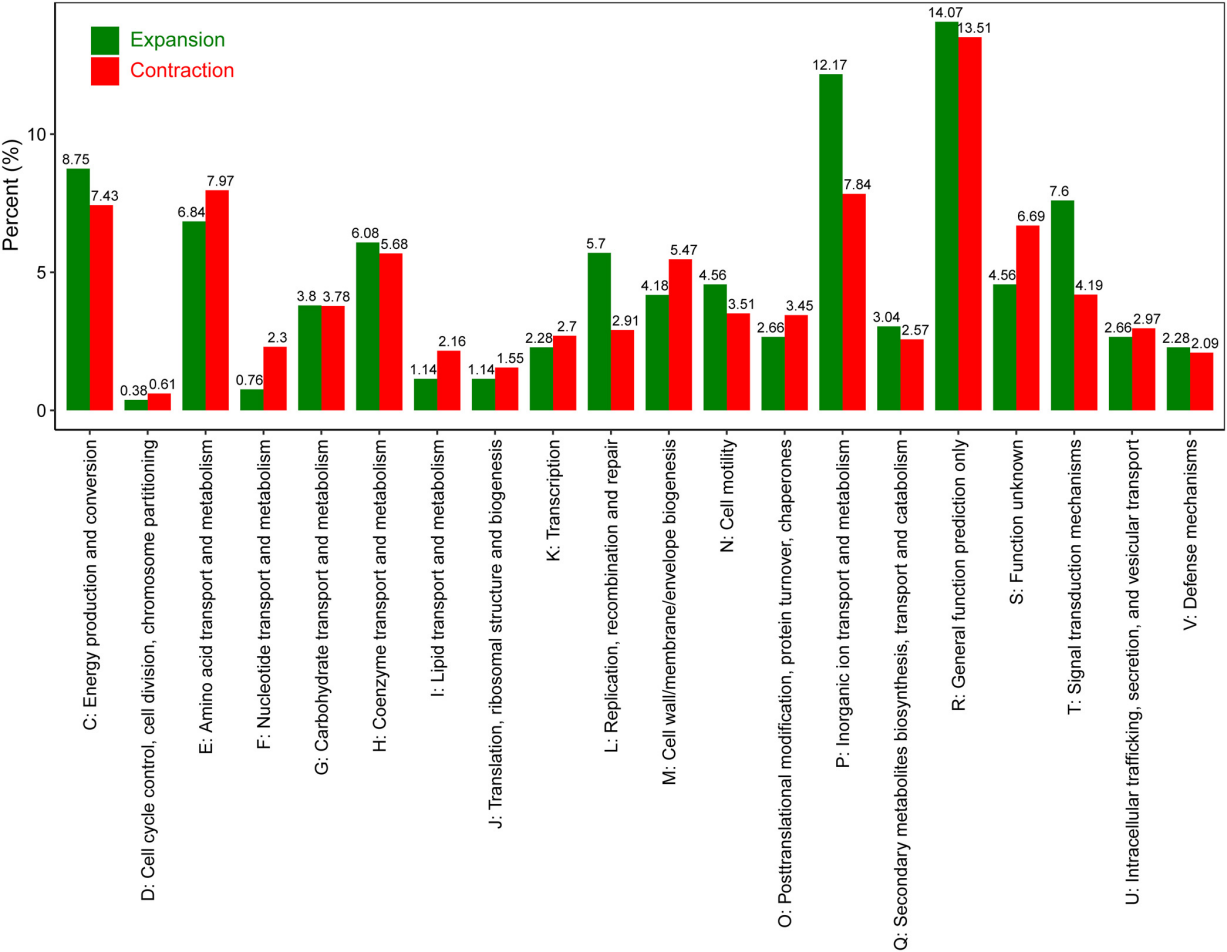
Distribution of COG categories in the Campylobacter lineage-specific expanded and contracted genome.

Moreover, we found potential clues to the genomic diversity of these Campylobacter species. Recent comparative genomic studies have demonstrated that genome expansion/contraction is a key factor leading to changes in the biological capabilities of bacteria (35). Previous findings for horizontal gene transfer analysis of 30 Campylobacter species identified some functional genes that were highly variable (36). Here, we used gene family expansion and contraction analysis based on the total gene family in the pan-genome and the phylogenetic tree to gain insight into the evolutionary flexibility and lineage-specific gene family expansion and contraction for 39 Campylobacter species. Reconstruction of genome-wide gene family expansion and contraction histories in 39 Campylobacter species revealed that 2,032 gene families were estimated to be present in the ancestral genome (Fig. 2). The evolutionary flexibility of these Campylobacter genomes was evident in determining the gene family expansion and contraction of each lineage. Numerically, there were 521 expansion events occurring on these terminal branches (lineage specific), involving 263 nonredundant expanded gene families. The COG annotations of these expanded gene families showed that “inorganic ion transport and metabolism” (P), “energy production and conversion” (C), and “signal transduction mechanisms” (T) demonstrated the largest gene family expansion. (Fig. 3). For terminal contraction events, of the 1,480 nonredundant gene families involved, the COG annotations of these contracted gene families were mainly related to “amino acid transport and metabolism” (E) and “inorganic ion transport and metabolism” (P) (Fig. 3).

### Presence-absence variation of virulence genes.

The virulence factors encoded by the pathogen genome enable the organism to manipulate host immune defenses, enhance a pathogen disease-inducing potential, and largely determine the outcome of infections (37). Thus, comprehensive knowledge of virulence factors is crucial to gain insights into the infection process. The pan-genome provided the opportunity to discover different pathogenic virulence genes present in diverse Campylobacter species and to explore the presence (or absence) information of all virulence genes among the Campylobacter species. We identified a total of 154 virulence genes in the 39 Campylobacter species, including 6 core genes, 147 accessory genes, and 1 unique gene. The virulence determinants of pathogenicity of these Campylobacter species include flagella, iron uptake, invasion, lipooligosaccharide (LOS), capsule, exotoxin, type IV secretion system (T4SS), and adherence.

**FIG 4:**
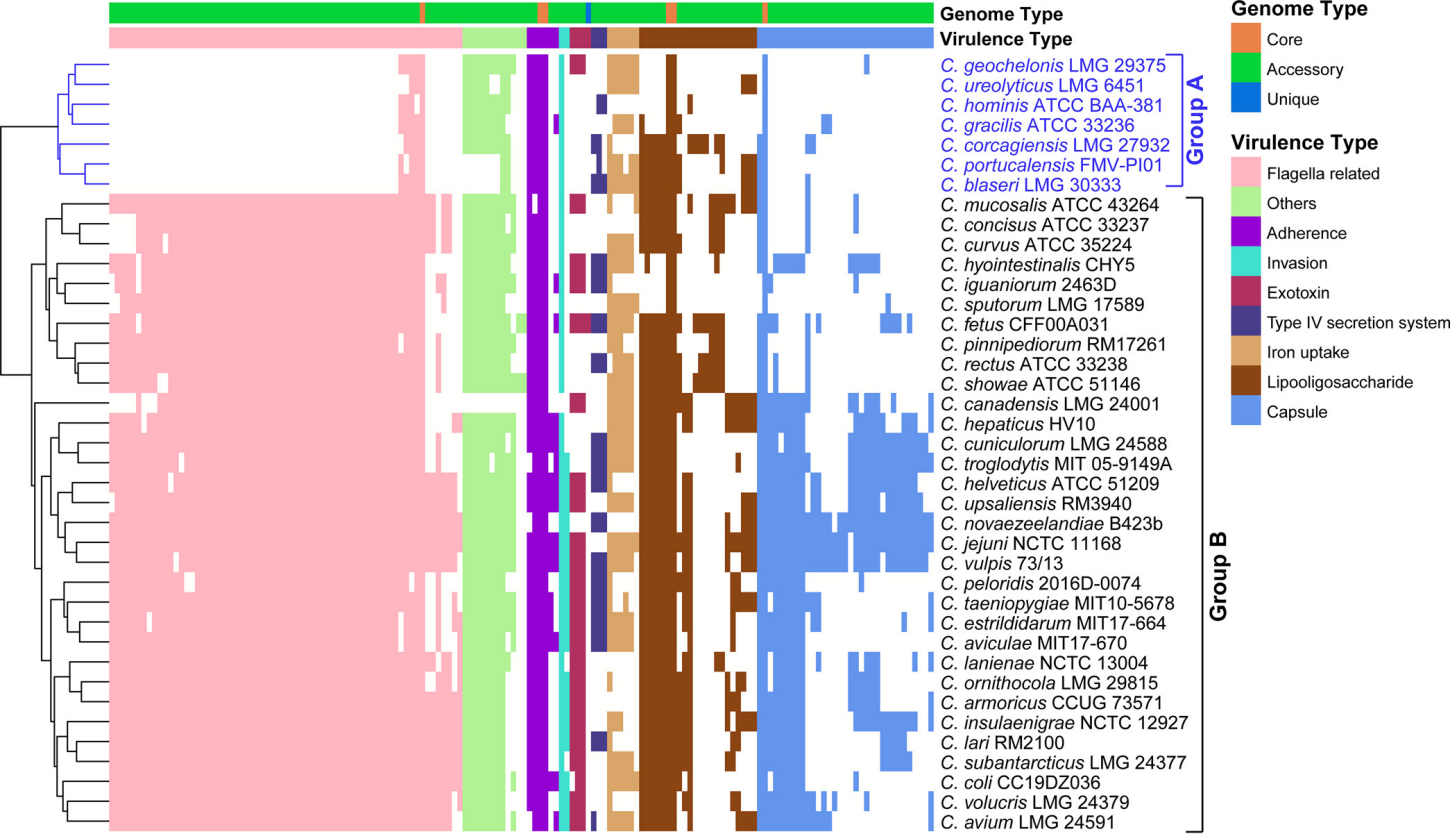
Presence (black)/absence (white) pattern of virulence factors in each Campylobacter genome. The tree on the left was clustered based on Euclidean distance.

Previous studies of Campylobacter have suggested that flagella confer darting motility, and these structures have long been recognized as being crucial to pathogenesis (38, 39). Some Campylobacter species, such as C. showae, have multiple flagella that move in a corkscrew pattern, whereas others, such as C. gracilis, are nonmotile (39). Flagella- and capsule-related genes represented the largest proportion of all virulence genes predicted in the Campylobacter pan-genome, accounting for 42.86% (66/154) and 21.43% (33/154), respectively, indicating their strong ability in colonization and immune evasion. The heatmap (Fig. 4) based on the presence and absence of all 154 virulence genes clearly showed that the distribution of virulence genes differed from each species. Due to the genomic heterogeneity, it is very likely that there will be fundamental functional variations between these species in terms of virulence functions. These species can be clustered into two main groups (Fig. 4) based on the distribution of virulence genes, with group A consisting of 7 species and group B consisting of the remaining 32 species. It was immediately apparent that members of group B possessed a block of flagella-related genes that were not found in group A. This block of genes is responsible for mediating motility, contributing to intestinal colonization, and playing a vital role in the pathogenesis of Campylobacter-mediated gastroenteritis (38). This indicated that the species of group B may acquire more genes associated with flagella, whereas more specialized group A species may not. Species in group A, such as C. ureolyticus, were aflagellate and were found to be less virulent than those with flagella, like C. jejuni (40). Previous studies by Piccirillo et al. (41–44) have reported that C. geochelonis and C. corcagiensis were motile, whereas C. hominis, C. gracilis, C. portucalensis, C. ureolyticus, and C. blaseri were straight rods lacking flagella and possessed no motile ability. *C. geochelonis* and *C. corcagiensis* also lacked flagella assembly genes, and the majority of cells were described as straight rods, with some being slightly curved (45). While group A members’ genomes contained genes encoding CheY, which interacts with the polar flagellar system, they lacked flagella assembly genes, such as *fliD*, *flgK*, or *flaB*. Therefore, the motility of *C. geochelonis* and *C. corcagiensis* may not be flagella dependent; not all motile prokaryotes use flagella, and other sensory systems are possible (46). More research is needed in the future to understand the mechanism of nonflagellar motility in *C. geochelonis* and *C. corcagiensis*.

**FIG 5:**
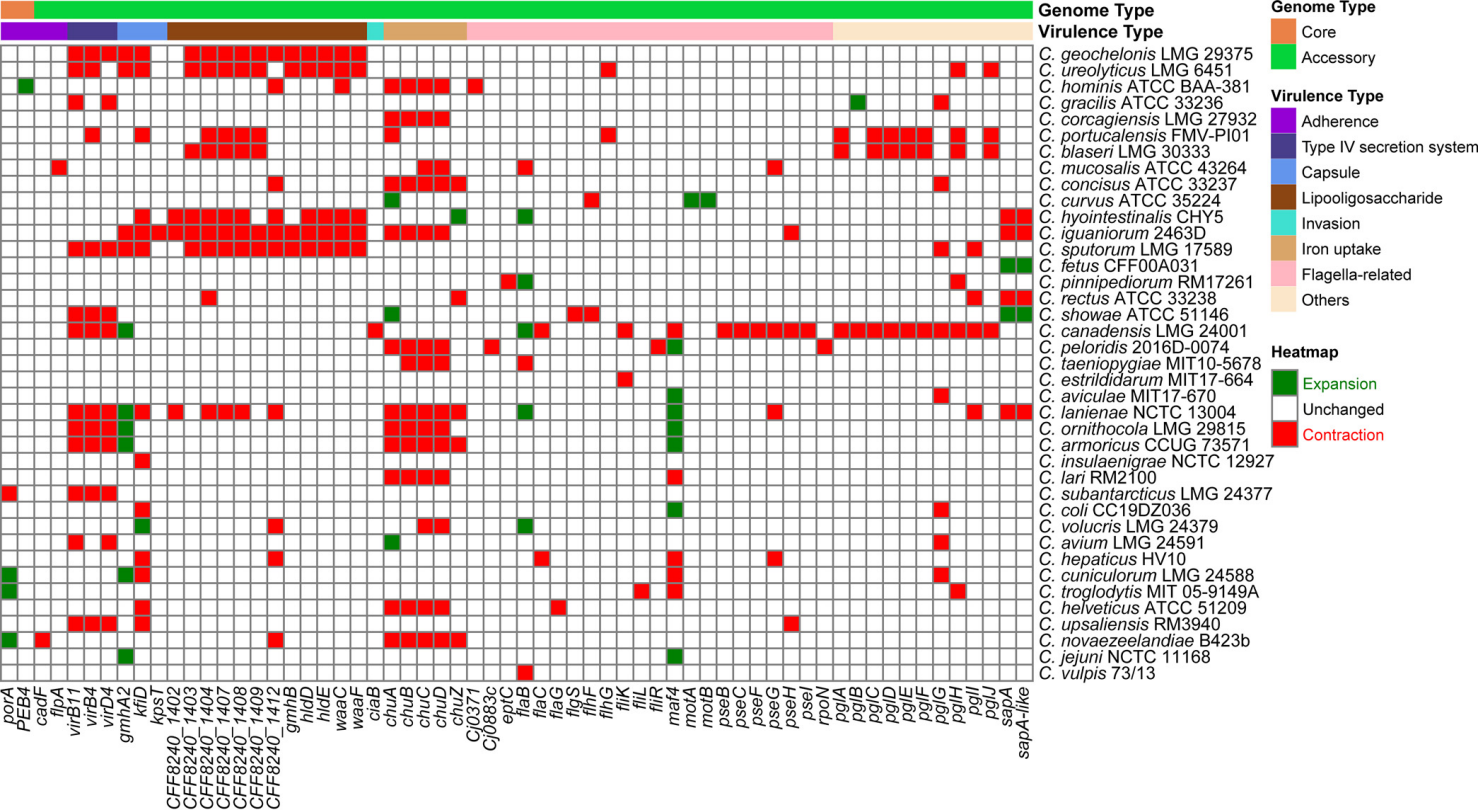
Expansion (green) and contraction (red) of the gene families encoding virulence factors in each Campylobacter species.

In contrast to the core genome, the accessory genome possessed more genes involved in critical activities of virulence, which contributed to further demonstrating the high degree of pathogenic heterogeneity between Campylobacter species. In the Campylobacter pan-genome, 6 core virulence genes (*porA*, *PEB4*, *cheY*, *htrB*, *Cj1135*, and *kpsF*) were shared by all 39 genomes, which are related to adherence (*porA* and *PEB4*), chemotaxis (*cheY*), and immune modulation (*htrB*, *Cj1135*, and *kpsF*), respectively. Among these core virulence genes, the gene *porA*, which encodes major outer membrane protein (MOMP), has been demonstrated to play a potential role in adherence associated with infections (47). This core gene was expanded in three species: C. cuniculorum LMG 24588, *C. troglodytis* MIT 05-9149A, and C. novaezealandiae B423b (Fig. 5). Another core gene *PEB4*, encoding major antigenic peptide PEB-cell binding factor, is related to enhanced adherence and biofilm formation and may contribute to the invasive behavior of Campylobacter (48). This *PEB4* gene in the C. hominis ATCC BAA-381 genome has experienced expansion. These 39 Campylobacter species all shared a conserved gene encoding CheY, one of the essential core chemotactic components that can interact with flagella to coordinate directional motions (49). In addition, CheY is one of the response regulators of two-component signal transduction systems, which aid bacteria in sensing and responding to their surroundings (50).

Lipooligosaccharide (LOS) is important for Campylobacter species to colonize a wide variety of hosts and intestinal niches, further contributing to their immune modulation (51). We identified 23 LOS-related genes in the Campylobacter pan-genome (Fig. S2), of which *Cj1135* encoding glucosyltransferase and *htrB* encoding lipid A biosynthesis lauroyl acyltransferase were both present in all 39 Campylobacter genomes. Many accessory genes related to LOS were also found to be constrained in several species, including *C. geochelonis* LMG 29375, C. ureolyticus LMG 6451, C. hyointestinalis CHY5, C. iguaniorum 2463D, and C. sputorum LMG 17589 (Fig. 5).

Cytolethal distending toxin (CDT) is reported to be one of the important elements required for the pathogenesis of pathogenic Campylobacter species (52). CDT can act as a genotoxin by causing damage and apoptosis to host cells, which is responsible for the occurrence and development of Campylobacter infection (52, 53). The three key genes of this toxin, *cdtA*, *cdtB*, and *cdtC*, are adjacent in the order of *cdtA*-*cdtB*-*cdtC* on the Campylobacter genomes. These three genes were present in the Campylobacter accessory genome (Fig. S3). In the genomes of 16 members of the Campylobacter genus, including C. portucalensis FMV-PI01, C. ureolyticus LMG 6451, C. curvus ATCC 35224, *C. showae* ATCC 51146, *C. sputorum* LMG 17589, C. pinnipediorum RM17261, *C. hepaticus* HV10, *C. gracilis* ATCC 33236, C. concisus ATCC 33237, C. corcagiensis LMG 27932, *C. novaezealandiae* B423b, *C. troglodytis* MIT 05-9149A, C. hominis ATCC BAA-381, *C. blaseri* LMG 30333, *C. cuniculorum* LMG 24588, and *C. rectus* ATCC 33238, these three genes (*cdtA*, *cdtB*, and *cdtC*) encoding CDT were absent, in agreement with the inability of *C. sputorum* LMG 17589 to cause disease in humans (16) and the finding that C. ureolyticus is less virulent than other Campylobacter species (such as C. jejuni) (40). These 16 species that lack the CDT-coding genes may be less toxic. The other 23 species seemed to be more pathogenic due to the presence of CDT-related genes.

### Presence-absence variation of antibiotic resistance genes.

**FIG 6:**
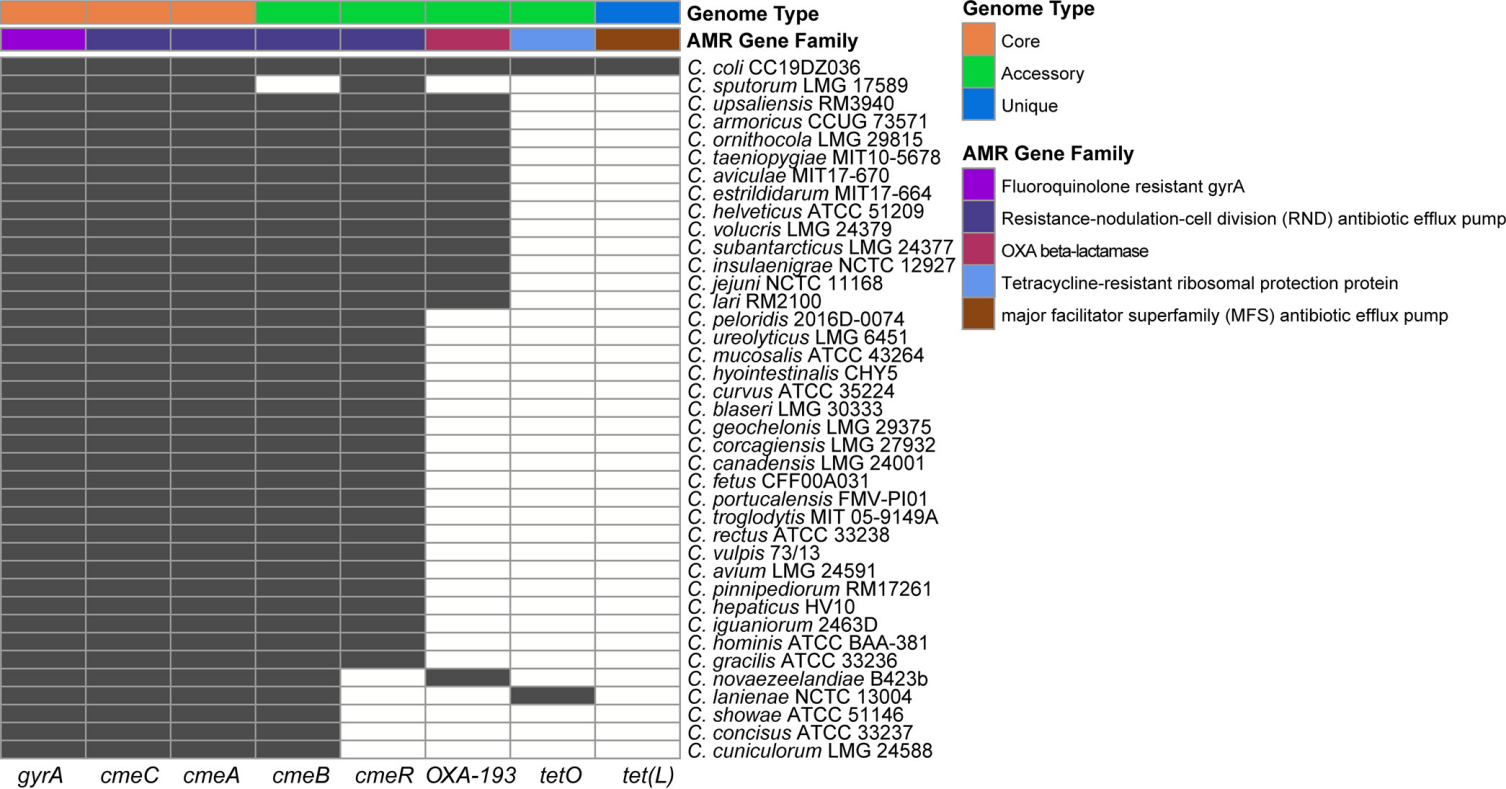
Presence (black)/absence (white) pattern of antibiotic resistance genes in each Campylobacter species.

Many Campylobacter members have been found to be resistant to multiple antibiotics, including tetracycline, erythromycin, ciprofloxacin, kanamycin, nalidixic acid, and chloramphenicol (54, 55). Human infections caused by antibiotic-resistant Campylobacter species complicate Campylobacter disease clinical management (56). We investigated the distribution of antibiotic resistance genes in the Campylobacter pan-genome to determine if they were susceptible to antibiotics. We found that all 39 Campylobacter genomes used in this study possessed the gene *gyrA*, encoding gyrase A, as well as *cmeA* and *cmeC*, encoding resistance-nodulation-cell division (RND) antibiotic efflux pump (Fig. 6). Mutations in C. jejuni *gyrA* have been reported to confer resistance to fluoroquinolones (57), and the variant of *cmeA* and *cmeC* led to enhanced resistance to cephalosporin, macrolide, fusidic acid, and fluoroquinolone (58). The prevalence of *gyrA*, *cmeA*, and *cmeC* genes in these species suggests that mutations of these genes may increase the resistance of these Campylobacter species to fluoroquinolone, cephalosporin, macrolide, and fusidic acid. The genes *cmeA*, *cmeB*, and *cmeC* involved in the multidrug efflux pump CmeABC are located in a cluster and are arranged in the order of *cmeA*-*cmeB*-*cmeC* in C. jejuni. This *cmeABC* cluster was conserved in all species except for *C. sputorum* LMG 17589 (Fig. 6). The transcriptional regulator *cmeR* of operon *cmeABC* was also adjacent to *cmeA* in *C. novaezealandiae* B423b and was absent in the genomes of C. lanienae NCTC 13004, *C. showae* ATCC 51146, *C. concisus* ATCC 33237, and *C. cuniculorum* LMG 24588. In addition, among these species, C. coli CC19DZ036 and *C. lanienae* NCTC 13004 carried the tetracycline resistance gene *tetO* (Fig. 6), which was reported to have synergistic effects with CmeABC (59). Moreover, we found that C. coli CC19DZ036 possessed more potential antibiotic resistance genes than other species, indicating that its antibiotic resistance may be more extensive.

### Conclusions.

In this study, we conducted a comparative pan-genome analysis of 39 Campylobacter species, revealing their pan-genome characteristics, genomic diversity, evolutionary relationships, and virulence profiles. This study provides a snapshot of the genomic diversity and evolution of different species that contribute to the metabolic and pathogenic diversity of 39 Campylobacter species.

The pan-genome of 39 Campylobacter members was found to be open and exhibited a high level of genomic variability, of which about 93.64% was variable. The COG analysis highlighted the adaptive functions of variable genes. The analyses of pan-genomes identified a set of universally conserved single-copy core genes, based on which a phylogenetic tree was constructed to confirm that *C. showae* ATCC 51146 and *C. rectus* ATCC 33238 represent more ancient lineages of Campylobacter. We also revealed that a number of genes have undergone expansion or contraction during the evolution of different Campylobacter species. These species have abundant virulence genes with functions that mirror pathogenicity differences, such as flagella and enterotoxin. The virulence genes expanded or contracted in each species emphasized the overall importance of gene expansion and contraction in shaping the variable part of the virulence genome. Furthermore, all studied Campylobacter species contained the *gyrA*, *cmeA*, and *cmeC* genes, variants of which have been reported to contribute to multidrug resistance. The virulence factors and antibiotic resistance genes characterized in this study would promote the effective use of candidates for the development of vaccines and antibiotics.

By examining the genomic variation within the Campylobacter genus, we highlighted how the process of gene expansion and contraction contributes to the gene content of the pan-genome and the virulence diversity within the Campylobacter genus, which will facilitate genetic studies and clinical research for Campylobacter.

## MATERIALS AND METHODS

### Public genomic resources.

For the pan-genome analysis, the genomic sequences for Campylobacter species, including the whole-genome sequences of 39 species that were previously developed, were downloaded from the Reference Sequence (RefSeq) database at NCBI (ftp://ftp.ncbi.nih.gov/genomes/, accessed on 10 July 2021). The genome-scale study used the most complete strain sampling of each Campylobacter species to date. We chose the genome with the highest assembly level and full-genome representation in the NCBI database when several sequences were available for a given species. The sequences considered in our study were from diverse sources, including humans, animals, and environments. Consequently, 39 Campylobacter genomes, which consisted of 32 complete genomes and 7 draft genomes, were carefully included in this study. Genome information, such as accession numbers, strain names, and genome statistics, is provided in Table S1 in the supplemental material.

### Pan-genome and core genome construction.

The genomes used in this study were first quality controlled using orthomclFilterFasta to filter out sequences with a length less than 10 and a proportion of stop codons greater than 20%. All the orthologous groups across 39 Campylobacter genomes were calculated using OrthoMCL (60) version 2.0.9 to identify homologous genes, including the core, accessory, and unique genomes. BLASTp searches were performed with a 1 × 10^−5^ E value cutoff, and the BLASTp results filtered by percent match length were set to 50%. The filtered BLASTp results were clustered by the Markov cluster (MCL) algorithm with inflation parameter 1.5, which has been widely used in other studies on microbial genomes to search for orthologs among multiple genomes.

### Phylogenetic analysis.

A single-copy core genome-based phylogenetic analysis of the 39 studied Campylobacter species was performed using PhyML (61) version 3.1. The substitution model, distribution of the gamma distribution shape parameter, and proportion of invariable sites were calculated using ProTest (62) version 3.4. For constructing the phylogenetic tree, the following options were used in PhyML software: maximum likelihood method, LG model for substitution model, 0.902 for gamma distribution shape parameter, 0.244 for the proportion of invariable sites, and 100 bootstrap replicates.

Divergence times between species were calculated using the MCMC tree program in PAML (63) (v4.9a). The program CAFÉ (64) (v3.1) was used to infer changes in the size of gene families.

### Functional analysis.

For the annotation of pan-genome genes with COG, a whole-genome BLASTp search was performed against the NCBI COG database with an E value cutoff of 1 × 10^−5^.

### Virulence factor analysis.

We subjected the proteins encoded by the genes in the pan-genome to a BLASTp search against the virulence factor database (VFDB) (65) to identify potential virulence-associated genes. The BLASTp search was performed using 90% identity, 90% coverage, and an E value cutoff of 1 × 10^−5^.

### Antibiotic resistance genes analysis.

To identify the antibiotic resistance genes in the Campylobacter pan-genome, genes were aligned against the comprehensive antibiotic resistance database (CARD) (66) using BLASTp with an E value cutoff of 1 × 10^−5^, a coverage cutoff of 90%, and an identity cutoff of 90%.

### Data availability.

All data generated or analyzed during this study are included in the manuscript and additional files.
