# Supplementary material for: Pan-Genome Analysis of Campylobacter: Insights on the Genomic Diversity and Virulence Profile
Source: Microbiol Spectr. 2022 Sep 7;10(5):e01029-22. doi: 10.1128/spectrum.01029-22 (PMC9602946; doi:10.1128/spectrum.01029-22)
Supplement: Supplemental file 1 — Supplemental material. Download spectrum.01029-22-s0001.pdf, PDF file, 1.5 MB [file spectrum.01029-22-s0001.pdf]

Supplementary file for "Pan-genome analysis of Campylobacter: Insights on the genomic diversity and virulence profile"

Chaofang Zhong[a], Bingpeng Qu[a], Kang Ning[b]#, Gang Hu[a]#

[a] College of Environmental and Life Sciences, Nanning Normal University, Nanning, Guangxi 530001, China

[b] Key Laboratory of Molecular Biophysics of the Ministry of Education, Hubei Key Laboratory of Bioinformatics and Molecular-imaging, Department of Bioinformatics and Systems Biology, College of Life Science and Technology, Huazhong University of Science and Technology, Wuhan, Hubei 430074, China

Running Head: Pan-genome study of *Campylobacter*

# Corresponding authors. E-mail: ahhugang@126.com, ningkang@hust.edu.cn

Supplementary Figures

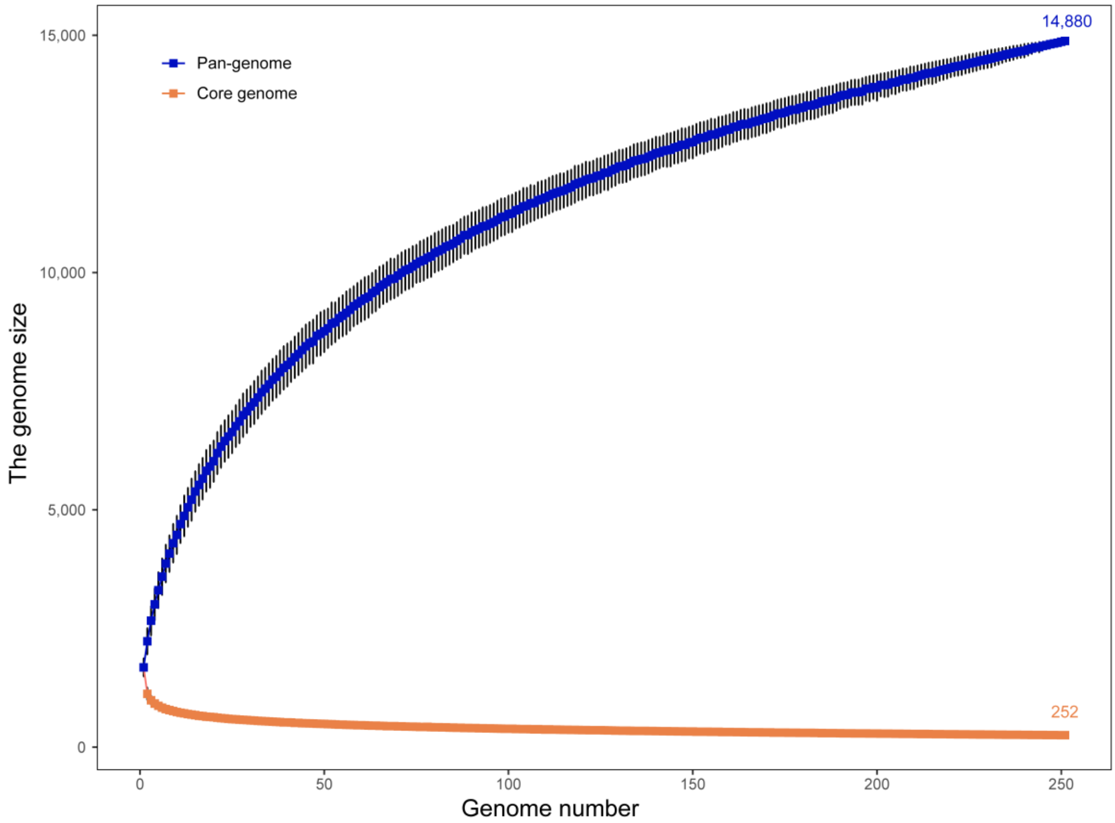

Supplementary Figure S1. The cumulative sizes for the pan-genome (blue) and core genome (orange) of 251 *Campylobacter* genomes.

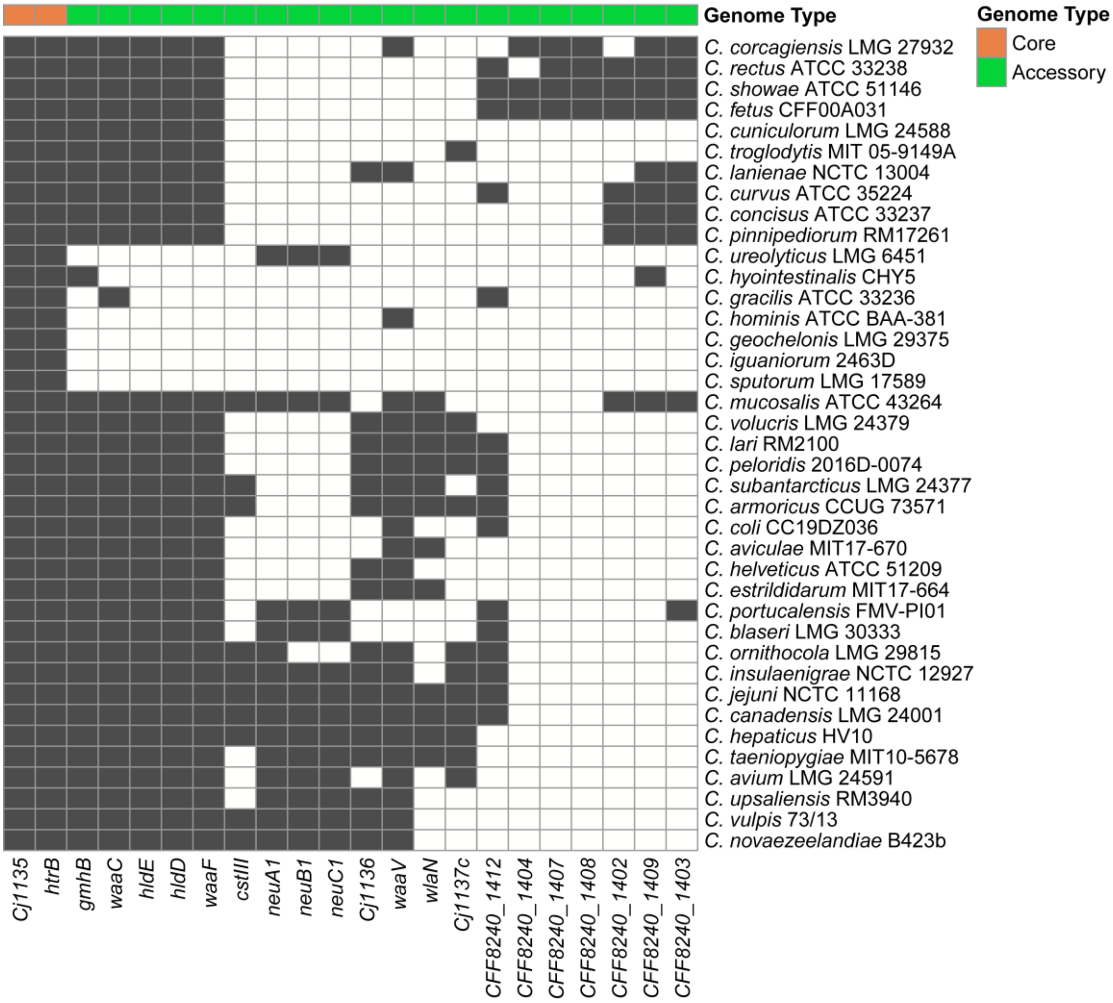

Supplementary Figure S2. The distribution pattern of Lipooligosaccharide encoded genes in 39 *Campylobacter* species.

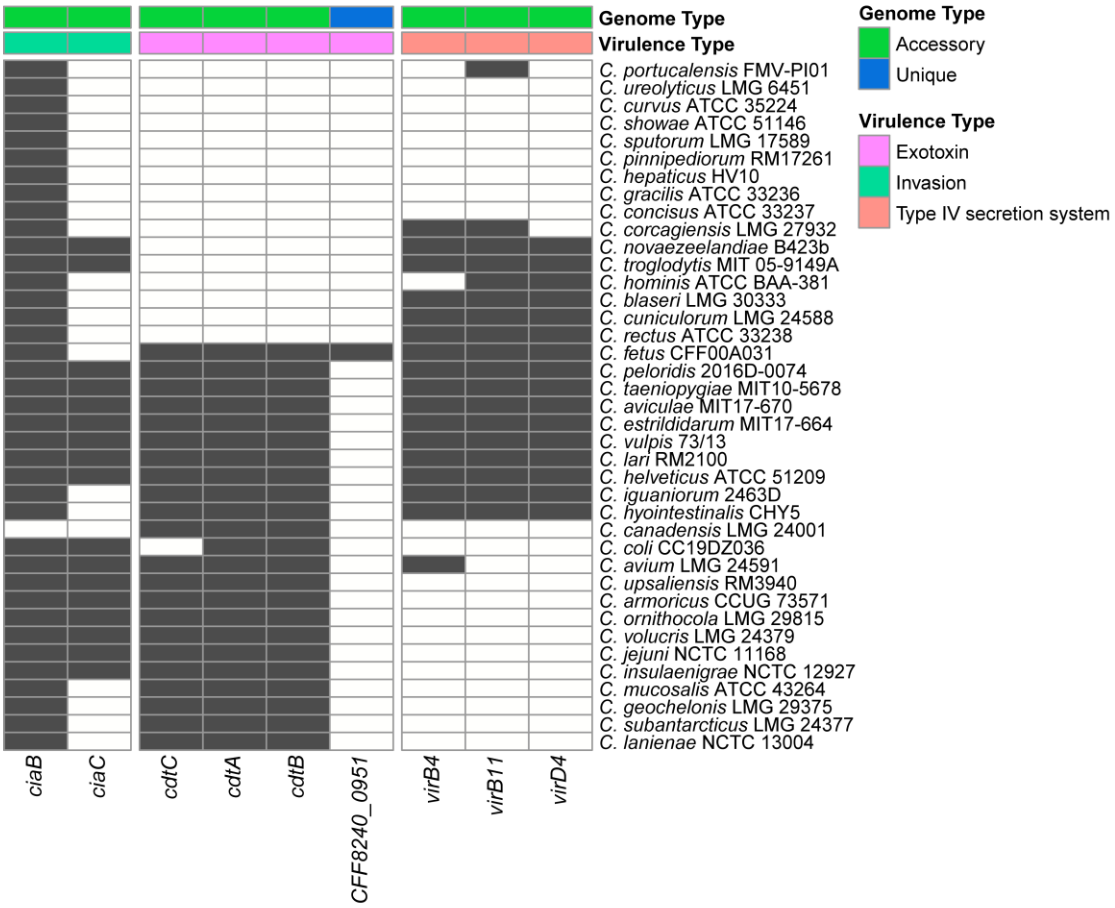

Supplementary Figure S3. The distribution pattern of virulence genes related to exotoxin, invasion, and type IV secretion system in 39 *Campylobacter* species.

Supplementary Tables

Supplementary Table S1. The general features of *Campylobacter* genomes used in this study.

Organism	Strain	Size (M)	GC%	CDS	RefSeq number	Assembly level	host	isolation source	geographic location	sources categories
Campylobacter armoricus	CCUG 73571	1.64	28.6	1,588	GCF_013372105.1	Complete Genome	-	River water	France: Brittany	environment
Campylobacter aviculae	MIT17-670	1.7	29.6	1,671	GCF_005406215.1	Contig	Taeniopygia guttata	Feces	USA	animal
Campylobacter avium	LMG 24591	1.74	34.2	1,699	GCF_002238335.1	Complete Genome	chicken	caecal	Italy: Bologna	animal
Campylobacter blaseri	LMG 30333	1.89	29.3	1,799	GCF_013201895.1	Complete Genome	Phoca vitulina	feces	Netherlands: Pieterburen	animal
Campylobacter canadensis	LMG 24001	1.92	27.4	1,788	GCF_013177655.1	Complete Genome	Whooping crane	cloacal swab	Canada: Calgary	animal
Campylobacter coli	CC19DZ036	1.76	31.4	1,736	GCF_018176575.1	Complete Genome	Duck	anal swab	China:Zhejiang	animal
Campylobacter concisus	ATCC 33237	1.84	37.6	1,782	GCF_001298465.1	Complete Genome	Homo sapiens	Gingival sulcus	USA	human
Campylobacter corcagiensis	LMG 27932	1.69	31.86	1,702	GCF_013201645.1	Complete Genome	Macaca silenus	feces	Ireland: Cork	animal
Campylobacter cuniculorum	LMG 24588	1.94	31.2	1,816	GCF_002104335.1	Complete Genome	Rabbit	caecal	Italy: Bologna	animal
Campylobacter curvus	ATCC 35224	1.97	44.3	1,849	GCF_013372125.1	Complete Genome	Homo sapiens	jaw abscess	USA: Massachusetts	human
Campylobacter estrildidarum	MIT17-664	1.66	29.2	1,772	GCF_005406205.1	Contig	Taeniopygia guttata	feces	USA	animal
Campylobacter fetus	CFF00A031	1.8	33.22	1,742	GCF_011600945.2	Complete Genome	bovine	preputial wash	Canada:British Columbia	animal
Campylobacter geochelonis	LMG 29375	2.17	33.5	1,900	GCF_013201685.1	Complete Genome	Testudo hermanni hermanni	-	Italy	animal
Campylobacter gracilis	ATCC 33236	2.28	46.6	2,106	GCF_001190745.1	Complete Genome	Homo sapiens	oral	USA	human
Campylobacter helveticus	ATCC 51209	1.87	34.34	1,892	GCF_002080395.1	Complete Genome	cat	feces	Switzerland: Berne	animal
Campylobacter hepaticus	HV10	1.52	28	1,395	GCF_001687475.2	Complete Genome	Gallus gallus	-	Australia: Victoria	animal
Campylobacter hominis	ATCC BAA-381	1.71	31.71	1,576	GCF_000017585.1	Complete Genome	Homo sapiens	feces	-	human
Campylobacter hyointestinalis	CHY5	1.81	33.29	1,791	GCF_013372165.1	Complete Genome	pig	stomach	-	animal
Campylobacter iguaniorum	2463D	1.81	35.7	1,863	GCF_001483985.1	Complete Genome	Iguana iguana	Iguana iguana	Netherlands	animal
Campylobacter insulaenigrae	NCTC 12927	1.47	28.2	1,444	GCF_000816185.1	Complete Genome	Marine mammal	Marine mammal	United Kingdom: Scotland	animal
Campylobacter jejuni	NCTC 11168	1.64	30.5	1,572	GCF_000009085.1	Complete Genome	Homo sapiens	feces	-	human
Campylobacter lanienae	NCTC 13004	1.59	34.6	1,582	GCF_002139935.1	Complete Genome	Homo sapiens	feces	Switzerland	human
Campylobacter lari	RM2100; ATCC BAA-1060D	1.57	29.54	1,592	GCF_000019205.1	Complete Genome	Homo sapiens	-	-	human
Campylobacter mucosalis	ATCC 43264	1.77	36.6	1,767	GCF_013372205.1	Complete Genome	Pig	small intestine	United Kingdom	animal
Campylobacter novaezeelandiae	B423b	1.57	27.4	1,548	GCF_004323845.1	Contig	Anas platyrhynchos	-	New Zealand	animal
Campylobacter ornithocola	LMG 29815	1.64	29.2	1,591	GCF_013201605.1	Complete Genome	Wild bird	feces	Chile: Valdivia	animal
Campylobacter peloridis	2016D-0074	1.73	28.54	1,613	GCF_014931075.1	Complete Genome	Shellfish	-	-	animal
Campylobacter pinnipediorum	RM17261	1.74	30.4	1,701	GCF_002021945.1	Complete Genome	sea lion	lung	USA: California	animal
Campylobacter portucalensis	FMV-PI01	1.77	28.3	1,774	GCF_009690845.1	Scaffold	bovine	preputial sample	Portugal: Lisbon	animal
Campylobacter rectus	ATCC 33238	2.57	44.7	2,246	GCF_004803795.1	Complete Genome	Homo sapiens	oral	USA: Boston	human
Campylobacter showae	ATCC 51146	2.1	45.7	1,952	GCF_004803815.1	Complete Genome	Homo sapiens	oral	Japan: Showa	human
Campylobacter sputorum	LMG 17589	1.73	29.6	1,702	GCF_002220755.1	Complete Genome	Homo sapiens	fecal	Canada: Ottawa	human
Campylobacter subantarcticus	LMG 24377	1.85	29.8	1,818	GCF_000816305.1	Complete Genome	-	Grey headed albatross	South Georgia and the South Sandwich Islands	animal
Campylobacter taeniopygiae	MIT10-5678	1.73	29.4	1,805	GCF_005406225.1	Contig	Taeniopygia guttata	feces	USA	animal
Campylobacter troglodytis	MIT 05-9149A	2.95	35.2	2,780	GCF_006864425.1	Contig	Chimpanzees	-	USA: MIT Cambridge, MA	animal
Campylobacter upsaliensis	RM3940	1.63	35.1	1,597	GCF_013372245.1	Complete Genome	Homo sapiens	fecal	USA: Los Angeles, California	human
Campylobacter ureolyticus	LMG 6451	1.82	29	1,742	GCF_013372225.1	Complete Genome	-	amniotic fluid	Canada: Alberta, Edmonton	-
Campylobacter volucris	LMG 24379	1.52	28.6	1,490	GCF_000816345.1	Complete Genome	Black headed gull	Black headed gull	Sweden	animal
Campylobacter vulpis	73/13	1.57	34.7	1,556	GCF_002738235.1	Scaffold	fox	blind gut	Italy: Monterenzio (Bologna)	animal
